# Network-based insights into miR-30a-5p-mediated regulation and EGCG targeting in triple-negative breast cancer

**DOI:** 10.3389/fbinf.2025.1735106

**Published:** 2025-12-19

**Authors:** Loganathan Chandramani Priya Dharshini, Abul Kalam Azad Mandal

**Affiliations:** Department of Biotechnology, School of Biosciences and Technology, Vellore Institute of Technology, Vellore, Tamil Nadu, India

**Keywords:** network pharmacology, TNBC, hsa-miR-30a-5p, EGCG, cell cycle regulation, molecular docking, molecular dynamics simulation

## Abstract

**Background:**

Triple-negative breast cancer (TNBC) is defined by the absence of ER, PR, and HER2 expression. This limits the targeted therapies, resulting in poor clinical outcomes. Identifying the molecular targets that can be regulated through miRNAs and natural compounds offers a potential therapeutic platform.

**Methods:**

We combined transcriptomic profiling with miRNA target prediction to identify genes regulated by miR-30a-5p and assess their interaction with the green tea polyphenol, epigallocatechin gallate (EGCG). Differentially expressed genes (DEGs) from TCGA-TNBC datasets and miRNA targets from miRDB, TargetScan, and miRTarBase were screened for common genes. Then, the protein-protein interaction and network topology analyses were performed to identify key hub genes. Molecular docking and simulation were carried out with the four key genes against EGCG.

**Results:**

Data integration yielded 393 overlapping genes and identified ten hub genes- *RRM2*, *KIF11*, *ANLN*, *CDC20*, *CCNA1*, *AGO2*, *YWHAZ*, *DTL*, *SKP2*, and *PCNA*. Pathway enrichment showed that all these hubs are involved in cell cycle and mitotic regulation, which was associated with poor TNBC prognosis. Mutation profiling revealed high alteration rates in *KIF11*, *ANLN, CDC20*, and *YWHAZ*, with increased missense mutations and C>T transitions. Molecular docking and simulations identified *YWHAZ* as the most favorable and structurally stable EGCG-binding target, compared to the other three key genes.

**Conclusion:**

The results emphasizes that EGCG has strong binding affinity towards YWHAZ, revealing that miR-30a-EGCG targets TNBC synergistically through cell-cycle-mediated pathways. The findings give rational support for miRNA-guided phytochemical-based TNBC therapeutic development.

## Graphical Abstract

**Figure:**
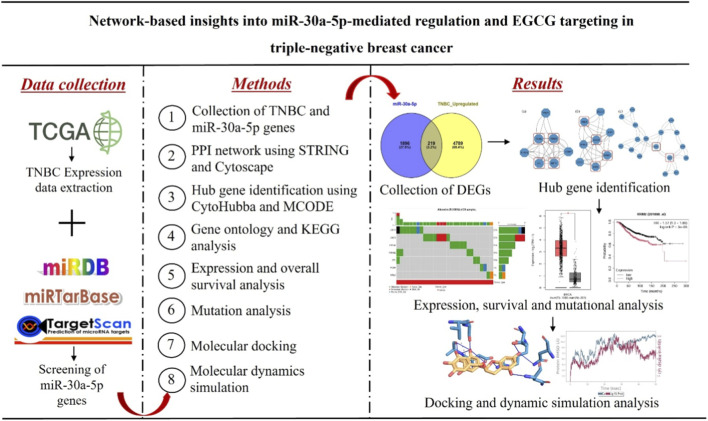
The overlapping genes between miR-30a-5p and TNBC were identified using network pharmacology. Docking and simulation showed that the YWHAZ-EGCG complex showed the highest stability and compactness, suggesting a synergistic therapeutic mechanism.

## 1 Introduction

Triple-negative breast cancer (TNBC), a molecularly diverse subtype of BC, accounts for 15%–20% of all BC cases (Wolff et al., 2013). It encompasses six subtypes and disproportionately affects premenopausal women (<40 years of age), mostly of African American and Hispanic descent from lower socioeconomic backgrounds (Morris et al., 2007). It is defined by heightened risk of early relapse, high metastatic potential, and dismal prognosis. After initial diagnosis, nearly half of all recurrences occur within 3–5 years, and patients under current treatment regimens have a median overall survival (OS) of only 10.2 months (Benotto et al., 2014). TNBC exhibits a distinct metastatic pattern, preferentially spreading to the brain, lungs, liver, and central nervous system (CNS) (Pradhan et al., 2023). The median time to recurrence ranges from 19 to 40 months, with a staggering 75% mortality rate within 3 months of recurrence (Yin et al., 2020). The distinct molecular phenotype and a lack of well-defined targets make TNBC resistant to endocrine and HER-2-based receptor-targeted therapies (Li et al., 2022). Although chemo-immunotherapy has shown efficacy in individuals exhibiting elevated programmed cell death ligand 1 (PD-L1) expression, major challenges such as therapeutic resistance, persistent metastatic burden, off-target toxicity, and acquired drug resistance continue to limit its success (Chaudhary et al., 2018). These difficulties emphasize the call for the identification of molecular targets and the development of targeted drug delivery to enhance patient outcomes.

MicroRNAs (miRNAs), approximately 19–23 nts, are short non-coding master regulators of post-transcriptional gene expression that drive numerous cellular processes from tumor initiation to metastasis. miRNAs that are dysregulated can drive oncogenesis through complex regulatory networks, eventually leading to therapy resistance (Kim and Croce, 2023). A known tumor suppressor is miR-30a, which inhibits proliferation, invasion, and migration while inducing apoptosis, thereby restraining tumor survival (Jiang et al., 2018). In TNBC, the expression of miR-30a is significantly decreased, correlating with higher histological grade and lymph node metastasis (Santana et al., 2023). Wang et al. (2024) observed that rosmarinic acid counteracts miR-30a-5p-mediated suppression of B-cell leukemia 2L11 (BCL2L11), increasing programmed cell death in MDA-MB-231-derived BC stem-like cells. Natural compounds are acquiring importance in oncological studies as sources of multi-target therapeutics. Green tea (*Camellia sinensis*) is highly known for its bioactive catechins, comprising 20%–30% of its dry weight (Chacko et al., 2010). Among which, EGCG is the most abundant and pharmacologically active catechin, exhibiting a broad anticancer effect (Aggarwal et al., 2022). It modulates several oncogenic pathways (PI3K/AKT, STAT3, and β-catenin), thereby inhibiting the nuclear translocation of their downstream effectors. This harmonized chaos induces apoptosis while suppressing the anti-apoptotic genes (Marin et al., 2023), fortifying its potential as a targeted anticancer agent.

High-throughput transcriptomic profiling provides a potent approach for unwinding the molecular level complexity of TNBC, allowing for the discovery of biomarkers, gene signatures, regulatory networks, and newer therapeutic targets (Supplit et al., 2021). Elango et al. (2023) reported CDC123 as a potential target of TNBC through transcriptomic and enrichment analyses, while Kaddoura et al. (2023) reported GATA3 and FOXA1 as important regulators within estrogen-dependent signaling pathways across multiple TNBC datasets.

In this study, we applied an integrative bioinformatics framework to identify miR-30a-5p-regulated molecular targets in TNBC. Furthermore, to explore the potential synergistic effect, we assessed the binding affinity of EGCG toward key hub genes via molecular docking, followed by molecular dynamics simulations (MDS) to evaluate the interaction dynamics and EGCG’s stability within the binding pockets. This multi-layered approach provides mechanistic insight into miRNA-phytochemical synergy and supports the rational development of multi-target therapeutic strategies of TNBC.

## 2 Materials and methods

### 2.1 Screening and processing the DEGs in TNBC

**FIGURE 1:**
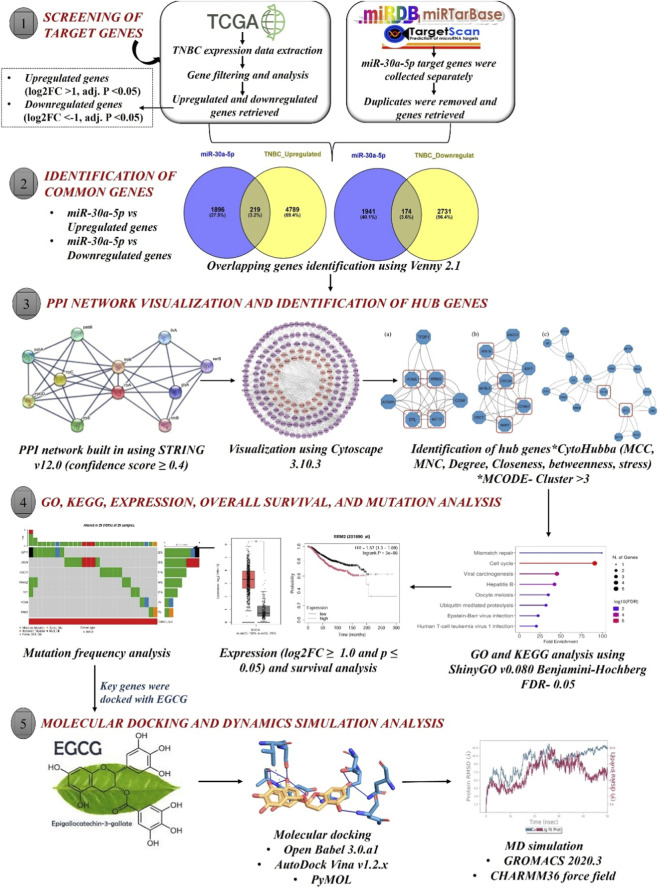
Graphical illustration depicting the overall experimental workflow.

To delineate differentially expressed genes (DEGs), RNASeq gene expression data of BRCA were retrieved from the TCGA database. Differential gene expression analysis was carried out using the R package “DESeq2”, and the Wald test was applied for significance testing. Then, the genes are ranked by adjusted *p-*values based on the Benjamini-Hochberg algorithm. Genes with log2 FC > 1 and adj. *p* < 0.05 were considered upregulated, while those with log2 FC < −1 and adj. *p* < 0.05 were considered downregulated genes. The functional annotation of the identified DEGs was conducted using the g:Profiler, a web-based interface (Cui et al., 2024; Zhao et al., 2022). Finally, visualization of DEGs was performed using the “ggplot2” package and presented as a volcano plot. This DEG profiling workflow and code were obtained from the methodology described by Krishnamoorthy and Karuppasamy (2024). The preprocessing pipeline for TNBC DEGs is available at the following GitHub repository (https://github.com/vitmilab/lab-projects/tree/main/DEG_Identification). The workflow of the study is depicted in Figure 1.

### 2.2 Selection of miR-30a-5p target genes

The experimentally verified targets of miR-30a-5p were obtained from databases such as miRTarBase, miRDB, and TargetScan. miRTarBase (https://ngdc.cncb.ac.cn/databasecommons/database/id/167) focuses exclusively on experimentally validated miRNA-target interactions supported by high-throughput evidence (Chen and Wang, 2020; Zhao et al., 2024). miRDB (https://mirdb.org/) employs the MirTarget computational tool to analyze miRNA-target interactions and their regulatory effects based on high-throughput sequencing (HTS) data (McGeary et al., 2019). TargetScan (https://www.targetscan.org/vert_80/) identifies potential miRNA targets by recognizing conserved motifs aligning the miRNA seed regions (Raudvere et al., 2019).

### 2.3 Acquisition of the common targets of miR-30a-5p and TNBC DEGs

The common targets between “upregulated DEGs vs. miR-30a-5p targets” and “downregulated DEGs vs. miR-30a-5p targets” were identified through “Venny 2.1 (https://bioinfogp.cnb.csic.es/tools/venny/) by entering the miR-30a-5p targets and disease genes in the query box (Oliveros, 2007).

### 2.4 PPI network construction

Protein-protein interactions (PPIs) among identified DEGs were assessed using the STRING database v12.0 (https://string-db.org/) (Scklarczyk et al., 2023; von Mering et al., 2004). Predicted interactions for the target genes were extracted using a medium confidence score ≥0.4, as calculated by STRING’s probabilistic scoring system (Equation 1). The confidence score shows the estimated likelihood that a predicted interaction reflects a true biological association, derived from combined evidence including experiments, co-expression, curated databases, and text mining. A score of ≥0.4 was selected to balance network connectivity and specificity, as using stricter cutoffs of ≥0.7 shattered the PPI network and removed valid interactions.

$$S=1-\prod_{i}\left(1-S_{i}\right)$$ (1)

### 2.5 Visualization of protein-interaction network (PIN) and key gene identification

Protein-interaction network (PIN) from STRING was imported and visualized through Cytoscape 3.10.3 (https://cytoscape.org/). Topological analysis was performed using the cytoHubba plugin to identify the top 10 hub genes, through six centrality measures, including maximal centrality clique (MCC), maximum neighbour component (MNC), degree, closeness, betweenness, and stress centralities (Equations 2–7) (Chin et al., 2014). For each gene, the ranks from all six algorithms were computed to obtain the mean rank, and genes with the highest composite scores were defined as hub genes.

$$MCC\;\left(v\right)=\sum_{C\in S\left(v\right)}\left(\left|C\right|-1\right)!$$ (2)

Here, the maximal clique’s collection containing v is represented by $S\left(v\right)$ , while the product of all positive integers less than $\left|C\right|$ is denoted by and $\left(\left|C\right|-1\right)$ . Absence of an edge between the neighbour node v , then $MCC\;\left(v\right)$ equals degree.

$$MNC\;\left(v\right)=\left|V\right.(\left.MC\left(V\right)\right|$$ (3)

In this case, the maximum connected component of the $G\left[N\left(V\right)\right]$ is denoted by $MC\left(V\right)$ and $G\left[N\left(V\right)\right]$ is the induced subgraph of G by $N\left(V\right)$ .

$$\mathrm{Deg}\;\left(v\right)=\left|N\left(V\right)\right|$$ (4)

Here, the node is represented by V and the collections of its neighbours are denoted by $N\left(V\right)$ .

$$Clo\;\left(v\right)=\sum_{w\epsilon v}\frac{1}{dist\;\left(v,w\right)}$$ (5)

$$BC\;\left(v\right)=\sum_{s\neq t\neq v\epsilon c\left(v\right)}\frac{\sigma_{st\;\left(v\right)}}{\sigma_{st\;}}$$ (6)

In this case, the number of shortest paths between nodes s and t is represented by $\sigma_{st}$ .

$$Str\;\left(v\right)=\sum_{s\neq t\neq v\epsilon c\left(v\right)}\sigma_{st}\left(v\right)$$ (7)

Here, the number of shortest paths from the node s to t is represented by $\sigma_{st}\left(v\right),$ which uses the node v .

Gene clusters that are tightly linked within the PIN were extracted from the Molecular Complex Detection (MCODE) plugin. Core targets identified by cytoHubba were cross-referenced with the MCODE clusters with scores >3, using default parameters, to further refine the selection of biologically significant genes (Shannon et al., 2003).

### 2.6 Gene ontology (GO) and pathway enrichment analysis

The biological significance and pathways associated with the key genes were determined using ShinyGO v0.80 (https://bioinformatics.sdstate.edu/go/). The significance was assessed using Benjamini-Hochberg false discovery rate (FDR) of 0.05 (Ge et al., 2019). Subsequently, the top 10 GO pathways involved in biological process (BP), molecular function (MF), and cellular component (CC), along with Kyoto Encyclopedia of Genes and Genomes (KEGG) pathways, were selected. Fold enrichment of each term was obtained directly from the ShinyGO enrichment output. Fold enrichment shows the overrepresented genes in a particular pathway and is calculated as observed/expected overlap $\left(k/E\right)$ , where k is the number of submitted genes found in the GO/KEGG term, and E is the expected overlap, which was calculated using the formula: $E=\frac{K}{N}\;x\;n$ , where n is the number of genes submitted (10 genes), K is the number of genes annotated to that term, and N is the background universe automatically selected by the enrichment tool.

### 2.7 Expression and survival analysis

The expression levels between TNBC vs. normal breast tissues were assessed using the Gene Expression Profiling Interactive Analysis 2 (GEPIA2) server (http://gepia2.cancer-pku.cn/#index) (Tang et al., 2019), with log2 FC ≥ 1.0 and p ≤ 0.05. Furthermore, through Kaplan-Meier (KM) plotter (https://kmplot.com/analysis/), the overall survival (OS) analysis in TNBC patients was evaluated.

### 2.8 Mutation analysis

The mutation frequency was analyzed through the Gene Set Cancer Analysis (GSCA) database (https://guolab.wchscu.cn/GSCA/#/). This database provides a comprehensive depot of genomic alterations, including single-nucleotide variants (SNVs), in a variety of cancer forms, including breast invasive carcinoma (BRCA). SNV data corresponding to the hub genes were extracted and evaluated to determine the mutation frequency and variant types observed in BRCA samples.

### 2.9 Molecular docking

The target proteins’ 3D structure was obtained from RCSB Protein Data Bank (https://www.rcsb.org/) in PDB format. AutoDock Tools (ADT) 1.5.7 was used for protein preparation, where water molecules, ligands, and heteroatoms were removed. Subsequently, polar and Kollman charges were added, and the processed structures were saved in PDBQT format. The ligand (EGCG) structure was downloaded from PubChem (CID: 65,064) in SDF format and converted to 3D using Open Babel 3.0.a1, and PDBQT files were generated using AutoDock Tools. A grid box was defined for each target protein to encompass the known active/binding site. Molecular docking was performed using AutoDock Vina v1.2.x. The output PDBQT files were converted and visualized using PyMOL to analyse interactions and binding conformations. The binding affinities (kcal/mol) of the top-ranked poses were recorded. Binding energy represents the predicted free energy of ligand-protein binding $\Delta G_{binding}$ , where more negative values indicate stronger and more favorable interactions. Using the PLIP server, the interactions (hydrogen bonding, π–π stacking, and hydrophobic interactions) were evaluated.

### 2.10 Molecular dynamics (MD) simulation

GROMACS 2020.3 package with the CHARMM36 force field was used for MD simulations to ascertain the structural stability and integrity of the four gene-ligand complexes (Van Der Spoel et al., 2005). Ligand parameter and topology files were generated through the CHARMM General Force Field (CGenF). Each protein-ligand complex was placed in a dodecahedron box using a simple point charge (SPC) water model, and the system’s neutrality was ensured by introducing three chlorine counterions. The system’s energy minimization was carried out using the steepest descent algorithm to eliminate unfavourable weak Van der Waals contacts. The algorithms, like the Particle Mesh Ewald (PME) method and the linear constraint solver (LINCS), were applied to constrain the electrostatic and hydrogen bond interactions. The system was equilibrated using canonical NVT (number of particles, volume, and temperature) and isobaric NPT (number of particles, pressure, and temperature) ensembles to stabilize its thermodynamic conditions. Berendsen thermostat used to heat the system to 300 K gradually, with a time lapse of 0.1 ps and a pressure of 1 bar. Then, the MD simulation was carried out for 100 ns with a 2-fs integration time step, and the trajectory data (RMSD, RMSF, Rg, and SASA) were analyzed based on the Formula (8-10). SASA is calculated from the atomic coordinates of a native protein numerically (Ghahremanian et al., 2022).

$$RMSD=\sqrt{\frac{1}{N}\sum_{i=1}^{N}{\left(x_{1}^{m}-x_{1}^{1}\right)}^{2}+{\left(y_{1}^{m}-y_{1}^{1}\right)}^{2}+{\left(z_{1}^{m}-z_{1}^{1}\right)}^{2}\;}$$ (8)

Where $x^{m}$ , $y^{m}$ , $z^{m}$ are the initial coordinates and $x^{1}$ , $y^{1}$ , $z^{1}$ are the trajectory coordinates at frame t, and N is the number of atoms.

$$RMSF=\sqrt{\frac{1}{T}\sum_{i=1}^{T}{\left(x_{i}-\bar{x}\right)}^{2}}$$ (9)

Where N is the trajectory frame numbers and $\bar{x}$ is the time-averaged position.

$$Rg=\sqrt{\frac{1}{N}{\sum_{i=1}^{N}\left|r\left(i\right)-r_{center}\right|}^{2}}$$ (10)

Where $r\left(i\right)$ is the coordinates of the atom i , and $r_{center}$ is the center of mass, and N is the number of protein atoms.

## 3 Results

### 3.1 Identification of DEGs

TNBC-specific transcriptomic data were obtained from the TCGA-BRCA dataset. The dataset was preprocessed, and the DEGs were identified using the “DESeq2 package” in R, ensuing an initial finding of 43,881 DEGs. Subsequently, the data were filtered based on log2 FC and adj. *p* value, resulting in a 5008 upregulated and 2,905 downregulated refined gene list within the TNBC dataset.

**FIGURE 2:**
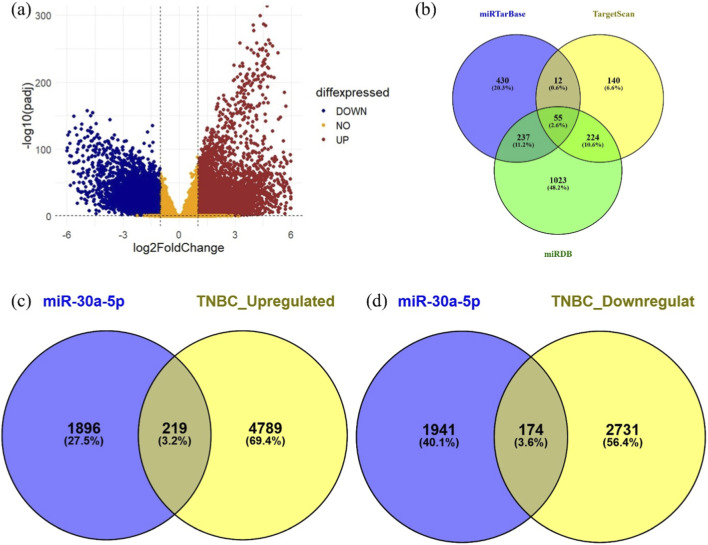
**(a)** Volcano plot showing differentially expressed genes of TNBC-BRCA; **(b)** 2,115 target genes from miRTarBase, TargetScan, and miRDB; Venn diagrams for overlapping genes related to **(c)** hsa-miR-30a-5p- TNBC upregulated genes, **(d)** hsa-miR-30a-5p- TNBC downregulated genes.

Functional annotation of these DEGs was performed using g:Profiler to traverse through their biological importance. Expression of DEGs was visually interpreted through a volcano plot (Figure 2a), markedly showing upregulated genes in red and downregulated genes in blue, giving a clear depiction of the expression landscape.

An integrated target gene set of 2,115 genes was compiled through the integrative curation of 2,704 hsa-miR-30a-5p-associated genes, sourced from three well-established miRNA target prediction databases: miRTarBase (734 genes), TargetScan (431 genes), and miRDB (1,539 genes) (Figure 2b). Redundancy was systematically eliminated to ensure maximal data fidelity in the gene set.

The convergence between miR-30a-5p target genes and TNBC-related genes was examined using InteractiVenn (Figures 2c,d). This integrative analysis revealed 393 overlapping genes, comprising 219 upregulated and 174 downregulated genes. All these genes are differentially expressed in TNBC and predicted to contain validated or putative miR-30a-5p binding. These represent functionally relevant targets of miR-30a-5p within the TNBC.

### 3.2 Hub gene identification from PPI network via CytoHubba and MCODE

The PPI analysis of the DEGs from the STRING database with a confidence score of ≥0.4 generated 392 nodes and 622 edges. The network exhibited an average node degree of 3.17 and an average local clustering coefficient of 0.351. Enrichment analysis yielded a highly significant PPI enrichment *p*-value of 2.49e-13 (*p <* 0.05), indicating that the observed interactions are unlikely to be random and reflect biologically meaningful associations.

**FIGURE 3:**
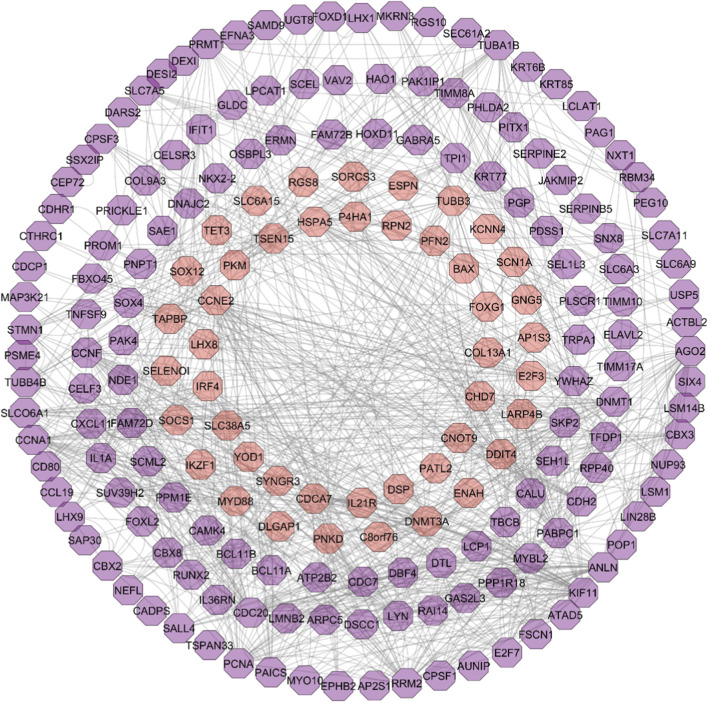
Visualization of protein-interaction network using Cytoscape. *Purple colour indicates the upregulated genes (219 genes) and orange indicates downregulated genes (174 genes)*.

The resulting PIN from STRING was imported into Cytoscape for further topological assessments (Figure 3). The network topology showed a clustering coefficient of 0.207 and a characteristic path length of 4.187, indicating that the network holds sturdy connections, toughness, and flexibility.

**FIGURE 4:**
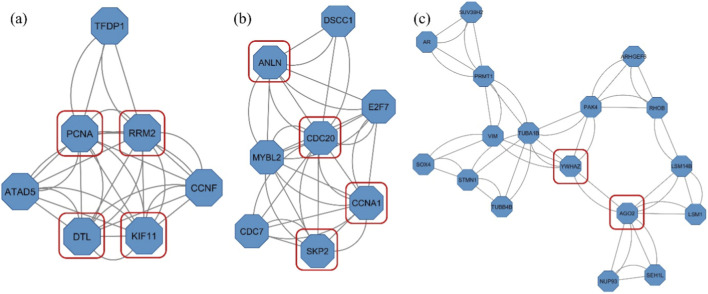
Gene clusters identified by MCODE with hub genes highlighted in red squares- **(a)** Cluster 1 (Score: 5.333), **(b)** Cluster 2 (Score: 4.857), and **(c)** Cluster 5 (Score: 3.125). *Clusters 3 and 4 did not contain any of the identified hub genes.*

CytoHubba plugin comprised the top 10 hub genes involved in TNBC survival and progression based on the ranking obtained from the six topological parameters. Furthermore, the MCODE plugin detected significant gene clusters within the PPI network. The clusters identified through MCODE were cross-referenced with the results from cytoHubba. Notably, *RRM2*, *KIF11*, *ANLN*, *CDC20*, *CCNA1*, *AGO2*, *YWHAZ*, *DTL*, *SKP2*, and *PCNA* consistently appeared across multiple cytoHubba rankings (Table 1) and were predominantly located within the MCODE clusters with a score >3 (Figure 4).

**TABLE 1:** Top 10 genes ranked by cytoHubba based on six topological parameters.

S.No	MCC	MNC	Degree	Closeness	Betweenness	Stress
1	*DTL* ^*^	*DTL* ^*^	*ANLN* ^*^	*CFL2*	*LYN*	*SNX1*
2	*RRM2* ^*^	*RRM2* ^*^	*RRM2* ^*^	*ANLN* ^*^	*ANLN* ^*^	*LEPR*
3	*MYBL2*	*ANLN* ^*^	*AGO2* ^*^	*VIM*	*AGO2* ^*^	*TIMM8A*
4	*E2F7*	*PCNA* ^*^	*CDC20* ^*^	*AR*	*CDC20* ^*^	*PCNA* ^*^
5	*CDC20* ^*^	*CDC20* ^*^	*YWHAZ* ^*^	*AGO2* ^*^	*YWHAZ* ^*^	*AGO2* ^*^
6	*KIF11* ^*^	*KIF11* ^*^	*KIF11* ^*^	*CDC20* ^*^	*ITSN1*	*YWHAZ* ^*^
7	*CDC7*	*CCNA1* ^*^	*CCNA1* ^*^	*YWHAZ* ^*^	*CDH2*	*CDH2*
8	*CCNA1* ^*^	*CDH2*	*CDH2*	*CDH2*	*BDNF*	*RPP40*
9	*CCNF*	*DNMT1*	*DNMT1*	*BDNF*	*DNMT1*	*BDNF*
10	*SKP2* ^*^	*SKP2* ^*^	*BDNF*	*DNMT1*	*RUNX2*	*DNMT1*

From the 12 topological parameters in cytoHubba- MCC, MNC, degree, Closeness, Betweenness, and Stress were selected to identify the most relevant hub genes (Repeated times ≥2). These shortlisted genes were further validated by cross-referencing with the gene cluster score >3 identified by the MCODE, plugin. Genes highlighted in * represent key hub genes identified through this integrative analysis.

### 3.3 Functional annotation through GO and KEGG

GO enrichment analysis was conducted using ShinyGO v0.80 to gain deeper insights into the biological significance of the ten key target genes regulated by miR-30a-5p in TNBC (Table 2). The GO BP analysis revealed significant enrichment of 437 pathways, with top-ranking terms including regulation of synapse maturation, mitotic cell cycle phase transition, regulation, processes, cell cycle, and its regulation. In the CC category, 67 enriched pathways were identified, with the hub genes predominantly localized to functionally critical structures and protein complexes. These included numerous complexes such as cyclin-dependent protein kinase (CDK) holoenzyme, cullin-RING ubiquitin ligase, transferase, intracellular protein-containing complex, microtubule cytoskeleton, nucleoplasm, and nuclear lumen. Within the MF, 80 significantly enriched terms were observed. The most prominent among these were purine-specific mismatch base pair DNA N-glycosylase activity, ribonucleotide-diphosphate reductase activity thioredoxin disulfide as acceptor, DNA polymerase processivity factor activity, ribonucleotide-diphosphate reductase activity, and enzyme activity associated with nucleotide metabolism and DNA replication. Primarily, the GO pathway enrichment analysis indicated that the genes are linked with cell cycle regulation, emphasizing the importance of hub genes in cell cycle regulation.

**TABLE 2:** Gene ontology (GO) analysis of the hub genes.

Pathways	No. of genes	Gene ID	Fold enrichment
Biological process			
Regulation of synapse maturation	2	*YWHAZ CDC20*	305.08
Mitotic cell cycle phase transition	6	*CCNA1 DTL SKP2 CDC20 RRM2 ANLN*	28.841
Regulation of mitotic cell cycle phase transition	4	*DTL CDC20 RRM2 ANLN*	25.636
Mitotic cell cycle process	8	*ANLN CCNA1 KIF11 DTL SKP2 CDC20 RRM2 PCNA*	22.187
Mitotic cell cycle	8	*ANLN CCNA1 KIF11 DTL SKP2 CDC20 RRM2 PCNA*	18.378
Regulation of cell cycle process	5	*DTL CDC20 RRM2 KIF11 ANLN*	14.648
Cell cycle process	8	*ANLN CCNA1 KIF11 DTL SKP2 CDC20 RRM2 PCNA*	13.856
Regulation of cell cycle	7	*CCNA1 DTL SKP2 CDC20 RRM2 KIF11 ANLN*	13.204
Cell cycle	8	*ANLN CCNA1 KIF11 DTL SKP2 CDC20 RRM2 PCNA*	9.450
Cellular component			
Cyclin-dependent protein kinase holoenzyme complex	2	*CCNA1 PCNA*	84.744
Cullin-RING ubiquitin ligase complex	3	*CDC20 SKP2 DTL*	36.127
Ubiquitin ligase complex	3	*CDC20 SKP2 DTL*	20.927
Transferase complex	5	*CDC20 CCNA1 SKP2 DTL PCNA*	12.462
Intracellular protein-containing complex	4	*CDC20 SKP2 AGO2 DTL*	10.353
Catalytic complex	7	*CDC20 CCNA1 SKP2 AGO2 DTL PCNA RRM2*	9.449
Microtubule cytoskeleton	5	*PCNA CCNA1 KIF11 DTL CDC20*	7.939
Cytoskeleton	6	*ANLN PCNA CCNA1 KIF11 DTL CDC20*	5.377
Nucleoplasm	8	*ANLN CDC20 PCNA DTL SKP2 AGO2 CCNA1 YWHAZ*	3.995
Nuclear lumen	8	*ANLN CDC20 PCNA DTL SKP2 AGO2 CCNA1 YWHAZ*	3.680
Molecular function			
Purine-specific mismatch base pair DNA N-glycosylase activity	1	*PCNA*	762.7
Ribonucleotide-diphosphate reductase activity thioredoxin disulfide as acceptor	1	*RRM2*	762.7
Oxidoreductase activity acting on CH and CH2 groups disulfide as acceptor	1	*RRM2*	762.7
DNA polymerase processivity factor activity	1	*PCNA*	762.7
Dinucleotide insertion or deletion binding	1	*PCNA*	762.7
Ribonucleotide-diphosphate reductase activity	1	*RRM2*	762.7
Ubiquitin ligase activator activity	1	*CDC20*	572.025
Protein C-terminus binding	3	*PCNA CDC20 AGO2*	33.814
Enzyme activity	5	*AGO2 CDC20 PCNA KIF11 YWHAZ*	5.114

From the KEGG analysis, out of 31 distinct pathways, the most significantly enriched ten pathways are detailed in Table 3. Hub genes are prominently involved in the cell cycle pathway, followed by viral carcinogenesis, mismatch repair, hepatitis B, and oocyte meiosis pathways. The dominant involvement of these genes in cell cycle-related pathways strongly suggests that miR-30a-5p may exert its therapeutic potential in TNBC by modulating core signaling cascades. The fold enrichment and total number of genes were reported in Tables 2, 3 for Gene Ontology and KEGG pathways, respectively.

**TABLE 3:** Top enriched pathways of the hub genes.

S. No	KEGG pathways	No. of genes	Gene id	Fold enrichment
1	Mismatch repair	1	*PCNA*	99.482
2	Cell cycle	5	*PCNA SKP2 YWHAZ CCNA1 CDC20*	90.797
3	Viral carcinogenesis	4	*SKP2 YWHAZ CCNA1 CDC20*	45.308
4	Hepatitis B	3	*PCNA YWHAZ CDC20*	42.372
5	Oocyte meiosis	2	*YWHAZ CDC20*	34.932
6	Ubiquitin mediated proteolysis	2	*SKP2 CDC20*	32.226
7	Epstein-Barr virus infection	2	*SKP2 CCNA1*	22.654
8	Human T-cell leukemia virus 1 infection	2	*CCNA1 CDC20*	20.613

### 3.4 Hub genes expression validation in TNBC

**FIGURE 5:**
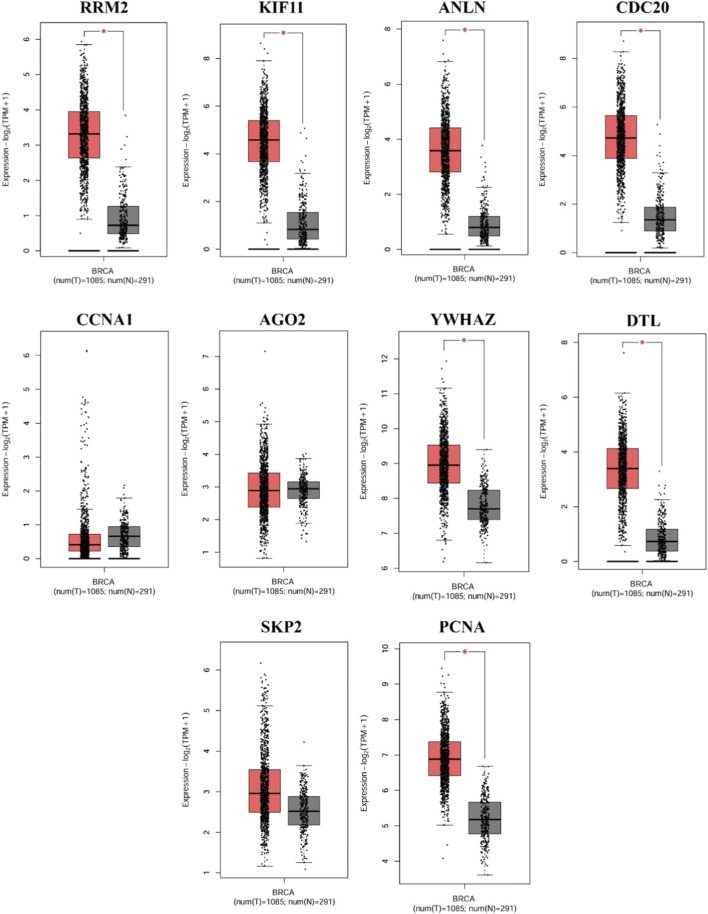
Expression validation of hub genes between normal and TNBC samples using GEPIA2. Box plots illustrate the expression levels of each hub gene in TNBC samples (left) compared to normal breast tissue (right) (* indicates *p* < 0.05).

The expression analysis of the ten hub genes was analyzed using GEPIA2 to assess their expression levels between normal and tumor samples. Expression levels were represented as -log_2_(TPM+1) to normalize the data. Among these, seven genes, namely, *RRM2*, *KIF11*, *ANLN*, *CDC20*, *YWHAZ*, *DTL*, and *PCNA*, exhibited significantly elevated expression in tumor samples (*p* ≤ 0.05). Whereas *CCNA1*, *AGO2*, and *SKP2* did not show statistically significant differences, indicating relatively stable expression between normal and tumor tissues (Figure 5).

### 3.5 Overall survival analysis

**FIGURE 6:**
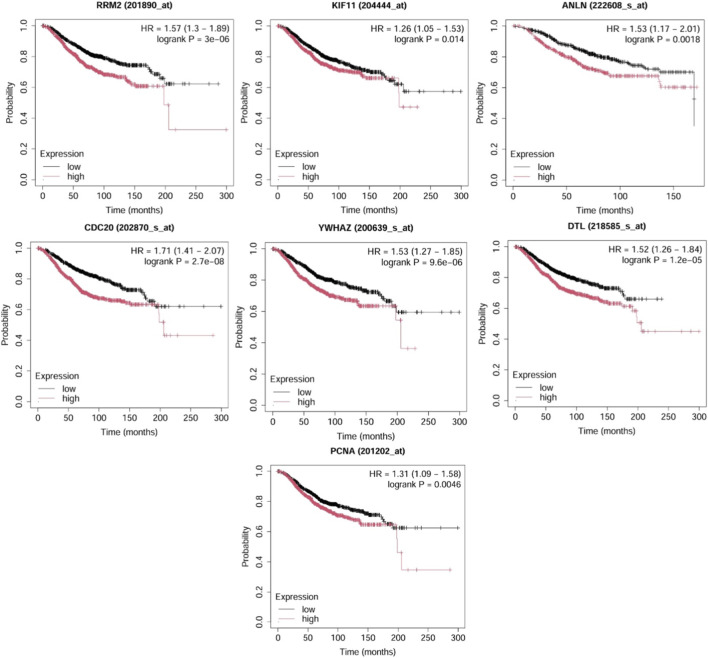
Overall survival analysis of the hub genes in TNBC patients using KM Plotter. High expression of each gene is associated with reduced overall survival, with *p* < 0.05 and HR > 1 considered statistically significant.

The prognostic significance of seven key upregulated hub genes, namely, *RRM2*, *KIF11*, *ANLN*, *CDC20*, *YWHAZ*, *DTL*, and *PCNA*, was evaluated for overall survival in TNBC patients using Kaplan-Meier survival analysis. From the above genes, *CDC20* exhibited the strongest prognostic effect with a hazard ratio of 1.71 (confidence interval (CI): 1.41–2.07; *p* = 2.7e-08). This was followed by *RRM2* (HR = 1.57, CI: 1.3–1.89, *p* = 3e-06), *YWHAZ* (HR = 1.53, *p* = 9.6e-06), *ANLN* (HR = 1.53, *p* = 0.0018), and *DTL* (HR = 1.52, *p* = 1.2e-05). Additionally, *PCNA* (HR = 1.31, *p* = 0.0046) and *KIF11* (HR = 1.26, *p* = 0.014) also exhibited significant associations with reduced survival probability (Figure 6).

### 3.6 Mutation analysis of the hub genes

**FIGURE 7:**
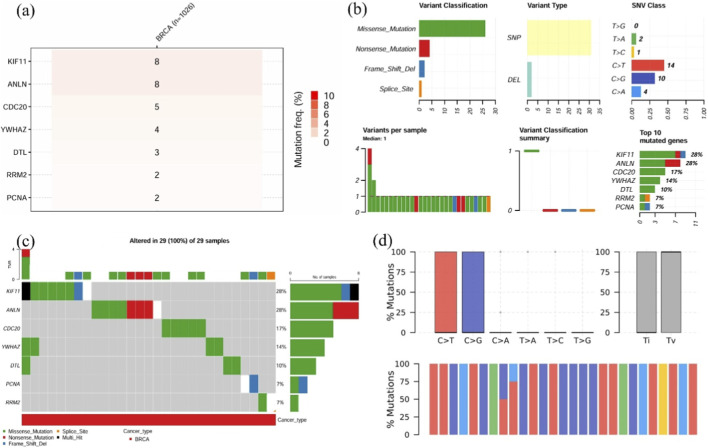
Mutation landscape of key hub genes in TNBC patients. **(a)** Heatmap showing the single-nucleotide variant (SNV) frequencies of hub genes across BRCA samples, **(b)** Mutation summary, including variant classifications, variant types, SNV classes, and the top 10 most frequently mutated genes in BRCA samples, **(c)** Bar plot illustrating the distribution of gene mutations across 29 samples, **(d)** Classification of mutations into transitions and transversions for the input genes.

Mutation analysis revealed that *KIF11* and *ANLN* had increased mutations (20% each), followed by *CDC20* (17%) and *YWHAZ* (14%). Missense mutation was found to be the abundant variant classification, with SNP and C>T as the variant type and SNV category, respectively. *CDC20* and *YWHAZ* carried missense mutations, whereas *ANLN* contained both missense and nonsense mutations. In contrast, frame-shift mutations were observed in *KIF11*, alongside missense and nonsense mutations (Figures 7a,b). Investigation of 29 BRCA samples showed that 100% of the samples displayed changes in at least one prognostic gene, with 28% of the cases in *KIF11* (Figure 7c). Moreover, comparable transitions and transversions with C>T and C>G being the most occurring conversions (Figure 7d). The increased burden of mutation diversity among the four prognostic genes suggests their role in disease progression and therapeutic targeting.

### 3.7 Molecular docking analysis

**FIGURE 8:**
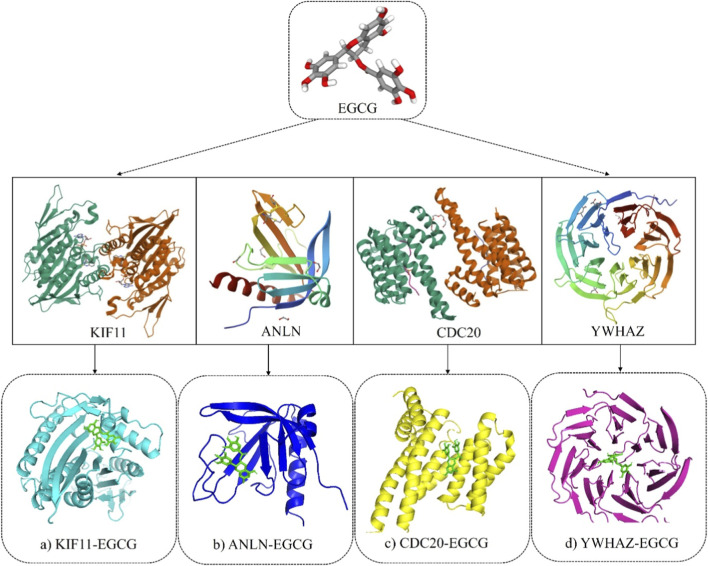
Molecular docking results of **(a)** KIF11-EGCG, **(b)** ANLN-EGCG, **(c)** CDC20-EGCG, and **(d)** YWHAZ-EGCG complexes.

EGCG exhibited the strongest affinity for YWHAZ (−10.1 kcal/mol), forming eight hydrogen (H_2_) bonds in the positions of Asp 191, Trp 234, Ser 275, Trp 276, Arg 316, Trp 363, Ser 404, and Ser 448. The next most favorable interaction was with ANLN (−8.9 kcal/mol), forming seven H_2_ bonds, followed by KIF11 (−8.5 kcal/mol) stabilized by eleven polar contacts. CDC20 showed the weakest binding of −7.0 kcal/mol with only four H_2_ bonds. Overall, the binding hierarchy was YWHAZ > ANLN > KIF11> CDC20, suggesting that YWHAZ have the most stable and complementary binding pocket for EGCG (Figure 8). Table 4 gives a summary of docking results with the number of hydrogen bonds.

**TABLE 4:** Summary of docking results showing the PDB ID of target proteins, predicted binding affinities (kcal/mol), number of hydrogen bonds, and key interacting residues for EGCG.

Gene name	PDB id	Binding affinity	No. of hydrogen bonds	Key interacting residues
KIF11	3K5E	−8.5	11	Asn 98, Asp 187, Asn 190, Arg 192, Val 194, Lys 260, Asn 262, Asp 322, Ser 323, Arg 327, Thr 328
ANLN	2Y7B	−8.9	7	Asn 1,033, Asn 1,036, Thr 1,060, Arg 1,062, Asp 1,068, Arg 1,069, Ser 1,074
CDC20	6F08	−7	4	Ser 45, Lys 120, Arg 127, Asn 173
YWHAZ	4GGC	−10.1	8	Asp 191, Trp 234, Ser 275, Trp 276, Arg 316, Trp 363, Ser 404, Ser 448

### 3.8 Molecular dynamics simulation

**FIGURE 9:**
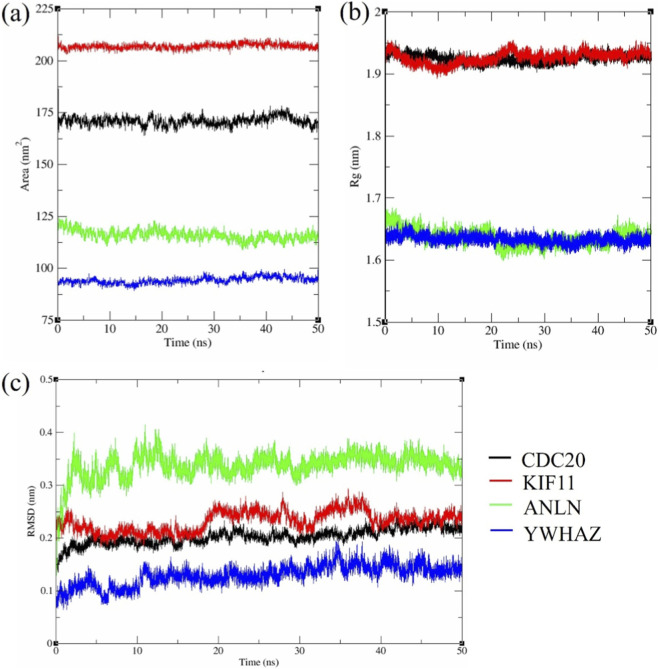
MD simulation analysis of KIF11, ANLN, CDC20, and YWHAZ with EGCG **(a)** RMSD, **(b)** SASA, **(c)** Radius of gyration.

The RMSD parameter (Figure 9a) examines the structural stability across all four protein-EGCG docked complexes. Notably, YWHAZ displayed good structural stability with slight deviation (⁓0.12 ± 0.02 nm), suggesting that the native scaffold was preserved by EGCG binding without persuading extensive conformational alterations. ANLN, in contrast, showed the highest fluctuations (⁓0.34 ± 0.03 nm), while CDC20 (⁓0.20 ± 0.03 nm) and KIF11 (⁓0.24 ± 0.03 nm) occupied intermediate positions. From the RMSD profile, it was observed that YWHAZ has a structurally rigid environment for EGCG binding, whereas CDC20 and KIF11 hold on to substantial backbone flexibility, leading to transitory shifts.

Consistent with the RMSD hierarchy, the solvent-accessible surface area (SASA) values (Figure 9b) revealed YWHAZ as the most compact complex (⁓95 ± 4 nm^2^), followed by ANLN (⁓120 ± 5 nm^2^), while CDC20 (⁓170 ± 5 nm^2^) and KIF11 (⁓205 ± 4 nm^2^) displayed substantially larger solvent exposure. The lower the SASA, the greater the compactness. YWHAZ shows a conformationally restrained state having low SASA, aligning with RMSD data. But CDC20 and KIF11 display greater flexibility and more solvent-exposed binding surfaces due to their increased SASA.

The Radius of Gyration (Rg) remained stable after rapid equilibrium within the first few ns (Figure 9c). Time-averaged Rg values strengthened the compactness hierarchy: YWHAZ (1.64 ± 0.02 nm) and ANLN (1.65 ± 0.02 nm) maintained tightly packed structures, whereas CDC20 (1.91 ± 0.02 nm) and KIF11 (1.93 ± 0.02 nm) were more extended. Low RMSD, SASA, and rigid Rg for YWHAZ highlight the increased stability, while it was vice-versa for CDC20 and KIF11 with their dynamic scaffolds.

**FIGURE 10:**
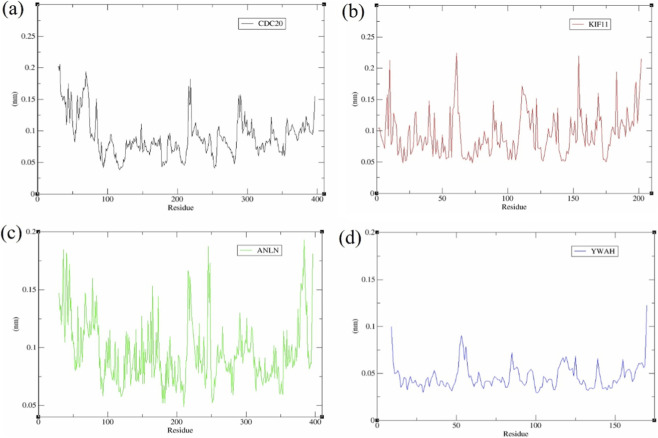
RMSF analysis of **(a)** CDC20, **(b)** KIF11, **(c)** ANLN, and **(d)** YWHAZ with EGCG.

RMSF demonstrated the structural grading of YWHAZ with the slightest mobility (baseline (⁓0.03–0.06 nm, peaks ⁓0.10–0.12 nm) (Figure 10d), reflecting a uniformly firm scaffold. ANLN showed intermediate flexibility (⁓0.12–0.18 nm at termini and loop regions) (Figure 10c), while CDC20 and KIF11 displayed distinct peaks (⁓0.20–0.27 nm) (Figures 10a,b), consistent with higher global flexibility.

## 4 Discussion

In recent years, TNBC’s pathological burden has accelerated significantly, defined by its aggressive phenotype, high histological grade, and rapid cellular proliferation. Unlike other subtypes of BC, TNBC lacks well-defined molecular targets, resulting in confined treatment options and contributing to poor clinical prognosis (Geyer et al., 2017). The “triple-negative” status of TNBC was defined through immunohistochemical (IHC) profiling reveals the absence of HER2 expression, with less than 1% ER and PR expression (Zagami and Carey, 2022). Researchers are nowadays gaining attention towards phytochemicals and microRNAs in cancer studies. A recent study by Yang et al. (2025) observed that CYCS and MYL12B as the potential targets of EGCG in nasopharyngeal carcinoma through IHC and docking approaches. Bioinformatics analysis demonstrated that EGCG affects NFκB1, Bcl-2, HIF-1α, MMP, etc., thereby reducing the risk of ovarian cancer (Xinqiang et al., 2017). MicroRNAs are widely known for post-transcriptional regulation, where miR-30a impacts prognosis associated with survival in ovarian cancer through hub gene regulation (Lu et al., 2020). Similarly, in osteosarcoma, miR-30a-3p suppresses tumor growth via PTEN upregulation (Zhong et al., 2017). All these analyses illustrate the power of integrative bioinformatics for focusing mechanistic studies.

Based on the above experimental evidence and challenges in TNBC treatment, there is a growing interest in combinatorial therapeutic approaches aimed at overcoming tumor heterogeneity and drug resistance. Our study combines EGCG and miR-30a-5p as a synergistic effect in TNBC treatment. EGCG, a bioactive polyphenol known for the suppression of cell cycle progression, apoptotic induction, and modulation of oncogenic pathways (Romano and Martel, 2021). Notably, miR-30a-5p enhanced the anticancer efficacy of small molecule drugs in BC by regulating gene expression involved in proliferation and survival (Jiang et al., 2018). Put together, EGCG and miR-30a provide a synergistic and potentially effective therapy for targeting several oncogenic pathways associated with TNBC.

In this study, transcriptomic profiling from the TCGA database yielded 5,008 upregulated and 2,905 downregulated TNBC genes. A total of 2,115 unique target genes of miR-30a-5p were identified through well-known miR-target prediction databases. Comparative intersection analysis uncovered 219 overlapping genes between miR-30a-5p targets and upregulated TNBC genes, and 174 overlapping genes with the downregulated set, yielding a refined pool of 393 common genes selected for further investigation. These genes were subjected to PPI network construction, resulting in a highly interconnected network of 392 nodes and 622 edges, visualized using Cytoscape. Network topology was examined using cytoHubba and MCODE plugins to identify key regulatory hubs. Intriguingly, *RRM2*, *KIF11*, *ANLN*, *CDC20*, *CCNA1*, *AGO2*, *YWHAZ*, *DTL*, *SKP2*, and *PCNA* emerged as top-ranked hub genes across multiple cytoHubba algorithms and were predominantly clustered within MCODE modules with scores exceeding 3. These findings underscore their potential as central molecular effectors in miR-30a-5p-mediated regulation and TNBC progression, providing valuable therapeutic targets.

Functional enrichment through GO and KEGG analyses demonstrated that the hub genes are involved in cell cycle processes, including their progression, regulatory mechanisms, the mitotic checkpoint complex, and the cyclin A2-CDK2 complex. These results collectively highlight the biological significance of the predicted target genes in maintaining cell cycle fidelity. Supporting this, Xiong et al. (2019) reported that upregulation of miR-30a-5p downregulated cyclin B1, cyclin D1, and c-myc, responsible for the cell cycle. Such evidence strongly suggests that miR-30a-5p can exert tumor-suppressive effects in TNBC by targeting essential regulators of the cell cycle pathway.

Expression analysis revealed that 7 out of 10 genes- *RRM2*, *KIF11*, *ANLN*, *CDC20*, *YWHAZ*, *DTL*, and *PCNA* were significantly upregulated in TNBC patients (*p* < 0.05). These genes are intricately involved in fundamental cellular pathways, including DNA replication, cell cycle regulation, and mitotic progression, thereby emphasizing their potential roles in sustaining the proliferative and aggressive phenotype of TNBC. Specifically, *RRM2*, *PCNA*, and *DTL* are associated with DNA synthesis and repair (Wilson et al., 2021; Zhang et al., 2021; Tian et al., 2024), while *KIF11* and *CDC20* regulate mitotic spindle formation and cell cycle transitions (Wang et al., 2025; Sitry-Shevah et al., 2024). *ANLN* is essential for cytokinesis (Maryam and Chin, 2021), and *YWHAZ* is implicated in modulating signal transduction pathways linked to cell survival (Pedroza et al., 2021). Further, in TNBC patients, the overall survival analysis revealed that elevated expression of the seven genes was linked with poorer outcomes (*p* < 0.05, HR > 1). Therefore, targeted regulation of these overexpressed oncogenic genes may effectively modulate critical tumorigenic pathways and improve patient prognosis. Mutation frequency analysis indicated that among the 7 identified hub genes, *KIF11*, *DTL*, *CDC20*, and *YWHAZ* exhibited the highest mutation rates, predominantly in the form of SNPs as the variant type. These genes emerged as the principal key hub genes within the study, stating that the gene modulation via miR-30a-5p may contribute to the downregulation of the key genes.

*KIF11* (kinesin family member 11 or kinesin-5 (E.g.,5)) is a vital ATP-driven motor protein that promotes bipolar spindle formation, which drives anti-parallel microtubules separation during cell division (Valensin et al., 2009). Overexpression of *KIF11* has been linked with poor clinical outcomes in numerous malignancies (Liu et al., 2016). In docetaxel-resistant TNBC cells, *KIF11* knockdown inhibited the proliferation of CD44+/CD24-cancer stem-like cells and removed their self-renewal capacity (Jiang et al., 2017). Similarly, *KIF11* inhibition has been shown to reduce breast cancer growth, migration, and invasion (Zhou et al., 2019).

*ANLN* (Anilin), an important cytokinesis regulator, has a dynamic subcellular localization. During interphase, it resides within the nucleus and relocates during telophase to the cytoplasm. This helps generate the formation of the contractile ring and development of the cleavage furrow through interactions with myosin, F-actin, RhoA, and septin (Piekny and Maddox, 2010). *ANLN* regulates proliferation through cell cycle modulation. Silencing *ANLN* in non-small cell lung cancer (NSCLC) and breast cancer cell lines resulted in polynucleated cells and markedly inhibited cell proliferation (Zhou et al., 2015).

*CDC20* (Cell Division Cycle 20), a crucial activator of the anaphase-promoting complex/cyclosome (APC/C), is necessary for appropriate mitotic progression. It is a potential therapeutic target, since dysregulation of its activity can result in mitotic abnormalities and tumorigenesis. Therefore, several *CDC20* inhibitors are currently under clinical investigation (Wang et al., 2015). While elevated *CDC20* expression has been found in BC (Karra et al., 2014), its function in TNBC is yet unknown. Song et al. (2021) observed that reduced *CDC20* expression showed a direct impact on cell migration and invasiveness in MDA-MB-231 cells.

*YWHAZ* (Tyrosine 3 monooxygenase/tryptophan 5-monooxygenase activation protein zeta or 14-3-3ζ), an important factor in signal transduction and tumor progression. Growing evidence suggests that *YWHAZ* is often overexpressed across numerous cancer types and acts as an oncogene by promoting cellular processes such as proliferation, migration, and invasion through interactions with ErbB2 and p85. Upstream microRNAs, such as miR-193b, miR-451, and miR-30c, control its expression (Gan et al., 2020; Wang et al., 2017). The importance of miRNA-mediated regulation was emphasized by a recent study that found that miR-136-5p elevation inhibited BC by directly targeting YWHAZ (Kong et al., 2023).

Docking results demonstrated that EGCG had a substantial binding affinity with YWHAZ (−10.1 kcal/mol), a validated target of miR-30a-5p, suggesting a potential synergistic interaction that could improve TNBC therapeutic efficacy. These results indicate the binding predictions, which require further experimental validation for functional confirmation. Previous literature stated that EGCG-protein binding energies typically range from −6 to −11.42 kcal/mol (Wang et al., 2019), consistent with our findings. For instance, Fanai et al. (2025) demonstrated the comparable binding affinity of EGCG with PPARGC1A (−9.55 kcal/mol), FOXO (−10.21 kcal/mol), and SIRT3 (−11.42 kcal/mol). Another study by Kamaraj (2025) showed the binding affinities of EGCG with ESR1 (−8.2 kcal/mol), MMP2 (−7.4 kcal/mol), MMP9 (−8.3 kcal/mol), MMP13 (−8.8 kcal/mol), and STAT1 (−7.3 kcal/mol). All the above evidence supports the biological plausibility of our modelled interactions. In line with the docking results, MD modelling showed that the YWHAZ-EGCG complex takes the most compact and stable structure. In contrast, ANLN displayed intermediate stabilization, and CDC20/KIF11 maintained dynamic solvent-exposed conformations (Paranthaman et al., 2025).

## 5 Conclusion

The present study integrates miRNA-based regulation with phytochemical intervention to target the risk caused by TNBC. By focusing on hsa-miR-30a-5p and EGCG, the study identified gene targets implicated in cell cycle and mitotic pathways, revealing multi-target therapeutic strategies. These findings provide support for co-delivery strategies using nanocarrier-based formulations, aiming to harness the synergistic effects of miRNAs and natural bioactive compounds that pave the way for newer treatment approaches for TNBC.

## Data Availability

The original contributions presented in the study are included in the article, further inquiries can be directed to the corresponding author.
